# Characterization of micronutrient supplements use by Brazilian children 6-59 months of age: *Brazilian National Survey on Child Nutrition* (ENANI-2019)

**DOI:** 10.1590/0102-311XEN085222

**Published:** 2023-08-28

**Authors:** Maiara Brusco de Freitas, Inês Rugani Ribeiro de Castro, Raquel Machado Schincaglia, Letícia B. Vertulli Carneiro, Nadya Helena Alves-Santos, Paula Normando, Pedro Gomes Andrade, Gilberto Kac, Brazilian Consortium on Child Nutrition 5

**Affiliations:** 1 Instituto de Nutrição Josué de Castro, Universidade Federal do Rio de Janeiro, Rio de Janeiro, Brasil.; 2 Instituto de Nutrição, Universidade do Estado do Rio de Janeiro, Rio de Janeiro, Brasil.; 3 Instituto de Estudos em Saúde Coletiva, Universidade Federal do Rio de Janeiro, Rio de Janeiro, Brasil.; 4 Instituto de Estudos em Saúde e Biológicas, Universidade Federal do Sul e Sudeste do Pará, Marabá, Brasil.

**Keywords:** Dietary Supplements, Vitamins, Minerals, Preschool Children, Suplementos Nutricionais, Vitaminas, Minerais, Pré-escolar, Suplementos Dietéticos, Vitaminas, Minerales, Preescolar

## Abstract

This study aimed to characterize micronutrient supplements use among Brazilian children 6-59 months of age included in the *Brazilian National Survey on Child Nutrition* (ENANI-2019; n = 12,598). Micronutrient supplements use at the time of the interview and the 6 months prior to it was evaluated using a structured questionnaire. The following indicators were included: micronutrient supplement use; supplements containing a single micronutrient; supplements of the Brazilian National Iron Supplementation Program (PNSF); multivitamin supplements with or without minerals; multivitamin supplements with minerals; multivitamin supplements without minerals. The estimates and their respective 95% confidence intervals (95%CI) were calculated for Brazil and according to macroregion, educational level of the mother or caregiver, and type of health care service used, considering the sampling plan, weights, and calibration. In Brazil, the prevalence of micronutrient supplements use was 54.2% (95%CI: 50.5; 57.8), with the highest prevalence in the North Region (80.2%; 95%CI: 74.9; 85.6) and among children 6-23 months of age (69.5%; 95%CI: 65.7; 73.3). The prevalence of the use of supplements containing exclusively iron and exclusively vitamin A in Brazil was 14.6% (95%CI: 13.1; 16.1) and 23.3% (95%CI: 19.4; 27.1), respectively. The prevalence of the use of multivitamin with or without minerals in Brazilian children 6-59 months of age was 24.3% (95%CI: 21.4; 27.2). These results may help to understand the practice of supplements use among Brazilian children and support the proposal of national public policies for the prevention and control of micronutrient deficiencies.

## Abstract (pt)

O objetivo deste estudo foi caracterizar o uso de suplementos de micronutrientes entre crianças brasileiras de 6-59 meses de idade incluídas no *Estudo Nacional de Alimentação e Nutrição Infantil* (ENANI-2019; n = 12.598). O uso de suplementos de micronutrientes no momento da entrevista e nos seis meses anteriores foi avaliado por meio de um questionário estruturado. Foram incluídos os seguintes indicadores: uso de suplemento de micronutrientes; suplementos contendo um único micronutriente; suplemento do Programa Nacional de Suplementação de Ferro (PNSF); suplementos multivitamínicos com ou sem minerais; suplementos multivitamínicos com minerais; suplementos multivitamínicos sem minerais. As estimativas pontuais e seus respectivos intervalos de 95% de confiança (IC95%) foram calculados para o Brasil e de acordo com a macrorregião, a escolaridade da mãe ou cuidadora e o tipo de serviço de saúde utilizado, considerando o plano, os pesos e a calibração amostral. No Brasil, a prevalência de uso de suplemento de micronutrientes foi de 54,2% (IC95%: 50,5; 57,8), com maior prevalência na Região Norte (80,2%; IC95%: 74,9; 85,6) e entre crianças de 6-23 meses de idade (69,5%; IC95%: 65,7; 73,3). A prevalência do uso de suplementos contendo apenas ferro e apenas vitamina A no Brasil foi de 14,6% (IC95%: 13,1; 16,1) e 23,3% (IC95%: 19,4; 27,1), respectivamente. A prevalência de uso de multivitamínicos com ou sem minerais em crianças brasileiras de 6-59 meses de idade foi de 24,3% (IC95%: 21,4; 27,2). Esses resultados podem auxiliar na compreensão da prática do uso de suplementos entre crianças brasileiras e apoiar a proposta de políticas públicas nacionais de prevenção e controle de deficiências de micronutrientes.

## Abstract (es)

El objetivo de este estudio fue caracterizar el uso de suplementos de micronutrientes entre niños brasileños con edades entre 6-59 meses incluidos en el *Estudio Nacional de Alimentación y Nutrición Infantil* (ENANI-2019; n = 12.598). El uso de suplementos de micronutrientes en el momento de la entrevista y en los seis meses anteriores se evaluó mediante un cuestionario estructurado. Se incluyeron los siguientes indicadores: uso de suplementos de micronutrientes; suplementos que contienen un solo micronutriente; suplemento del Programa Nacional de Suplementación con Hierro (PNSF); suplementos multivitamínicos con o sin minerales; suplementos multivitamínicos con minerales; suplementos multivitamínicos libres de minerales. Se calcularon las estimaciones puntuales para Brasil y sus respectivos intervalos del 95% de confianza (IC95%) de acuerdo con la macrorregión, el nivel educativo de la madre/cuidador y el tipo de servicio de salud utilizado, considerando el plan, los pesos y la calibración de la muestra. En Brasil, la prevalencia del uso de suplementos de micronutrientes fue del 54,2% (IC95%: 50,5; 57,8), con mayor prevalencia en la Región Norte (80,2%; IC95%: 74,9; 85,6) y entre niños con edades entre 6-23 meses (69,5%; IC95%: 65,7; 73,3). Las prevalencias del uso de suplementos que contienen solo hierro o solo vitamina A en Brasil fueron del 14,6% (IC95%: 13,1; 16,1) y del 23,3% (IC95%: 19,4; 27,1), respectivamente. La prevalencia de uso de multivitamínicos con o sin minerales en niños brasileños de 6-59 meses de edad fue del 24,3% (IC95%: 21,4; 27,2). Estos resultados pueden ayudar a comprender la práctica de uso de suplementos entre los niños brasileños y apoyar la propuesta de políticas públicas para la prevención y control de la carencia de micronutrientes.

## Introduction

Micronutrients are essential for adequate cellular metabolism and other organic functions ^1^. Their deficiency may harm the individual’s health, such as changes in linear growth ^2^, cognitive function ^3^, and immune system impairment ^4^.

Micronutrient deficiency is more prevalent in vulnerable populations, such as children < 5 years old ^5^, and affects more than 340 million children worldwide ^1^. Iron, vitamin A, zinc, iodine, and folate are among the most prevalent deficiencies in childhood and are greatly relevant for public health ^6^ ^,^ ^7^. Most outcomes caused by these deficiencies are reversible, with an adequate supply of micronutrients. However, some disorders may be permanent, depending on the severity, duration of the disability, and the stage of life in which they occur ^8^.

In Brazil, several public policies aim to prevent and control micronutrient deficiencies in children. Among them, there are supplementation programs with one or more micronutrients such as iron (Brazilian National Iron Supplementation Program - PNSF) ^9^, a powder mixture of 15 vitamins and minerals (Brazilian National Strategy of Fortification of Infant Feeding with Micronutrients in Powder - NutriSUS) ^9^, megadoses of vitamin A (Brazilian National Vitamin A Supplementation Program - PNSVA) ^9^, iron and folic acid fortification of wheat and maize flours ^10^, and iodine fortification of salt (Brazilian Program for Prevention and Control of Disorders due to Iodine Deficiency - Pró-Iodo) ^11^. Besides these public policies, the food industry fortifies many ultra-processed foods aimed at child nutrition with vitamins and minerals ^12^, and the pharmaceutical industry provides a wide variety of micronutrient supplements targeting the child population ^13^ ^,^ ^14^. In addition, the Pediatric Society encourages the use of some supplements, which are not always in line with the recommendations of the Brazilian Ministry of Health ^15^ ^,^ ^16^ ^,^ ^17^.

Data on the micronutrient supplements use are scarce and conflicting in Brazil. Local studies have reported an use prevalence ranging from 3% to 6% among individuals 0-14 years old ^18^ ^,^ ^19^ ^,^ ^20^ ^,^ ^21^. In the *Brazilian National Survey of Demography and Health* (PNDS) conducted in 2006, 39.6% of the children 6-11 months of age and 42.6% of those 12-23 months of age had consumed iron supplements 6 months before the interview. Also, 28.3% of children < 5 years old had consumed vitamin A supplements ^22^. In a Brazilian local study with children ≥ 6 months of age, the intake was more frequent in children born to women ≥ 25 years old (27.2%) and in those who were breastfed for less time ^21^.

Micronutrient supplements use has been prescribed by healthcare professionals for the maintenance and/or improvement of health ^23^ ^,^ ^24^. However, the exploitation of the image of micronutrient supplements as health-promoting products should be questioned, as they may increase the risk of nutrient use above safe levels ^21^ ^,^ ^23^. Thus, this study aims to characterize the use of micronutrient supplements, including those recommended by the Brazilian Ministry of Health, among Brazilian children 6-59 months of age, according to macroregion, education level of the mother or caregiver, and type of health care service used.

## Methods

### Study design and population

The *Brazilian National Survey on Child Nutrition* (ENANI-2019) is a population-based household survey that evaluates the feeding practices and anthropometric and micronutrient nutritional status of children < 5 years old ^25^. The ENANI-2019 presents complex probabilistic sampling, geographic stratification by macroregion, conglomeration by census tracts, and weight calibration. The details about the sample design are available in Vasconcellos et al. ^26^.

Data were collected from February 2019 to March 2020, and the sample comprised 14,558 children < 5 years old in 12,524 households distributed in 123 Brazilian municipalities. This study included children 6-59 months of age (n = 12,598) because micronutrient supplementation programs enroll only children ≥ 6 months of age.

### Questionnaire for evaluation of the use micronutrient supplements

During the interview, the mothers or caregivers of the children were asked about the micronutrient supplements use at the time of the interview and in the 6 months preceding it ^27^, including the use of NutriSUS and vitamin A megadose distributed through the Brazilian Unified National Health System (SUS). The questionnaire ^25^ included a list based on the main vitamin and mineral supplements available in Brazilian drugstores and the SUS as well as those mentioned in a survey conducted in Rio de Janeiro ^28^. There was also the possibility of including other supplements. For each supplement, the following data were recorded: quantity and frequency of use, motivation for use, who had prescribed the supplement, and where the product had been purchased. These variables will be analyzed in future publications.

The Brazilian Health Regulatory Agency (Anvisa) ^29^ classifies vitamin and mineral supplements as those products whose nutrient concentration does not reach 100% of the Dietary Reference Intake (DRI) and, therefore, are classified as low-risk. Conversely, those supplements that exceed 100% of the DRI are considered medications and thus require registration and package inserts ^29^. In this study, we evaluated both product categories as “micronutrient supplements”.

### Database organization

The microdata of ENANI-2019 underwent review and correction such as the exclusion of products that were not supplements or medications (e.g.: analgesics or not composed of vitamins and minerals, phytotherapeutic agents, probiotics); evaluation of the amounts of micronutrients in supplements and exclusion of excessive values; standardization of names with possible typos for those not listed in the questionnaire; research on the composition, dosage, and presentation of all micronutrient supplements; and analysis of the consistency and correction of inconsistent data. After this step, automatic imputation was used for implausible/inconsistent cases, missing data, and “do not know/did not want to answer” responses. Two methods were used: sequential hot deck implemented in the CSPro program (https://www.census.gov/data/software/cspro.html) and deterministic imputation ^30^. The choice of donors considered demographic, socioeconomic, and individual factors potentially associated with the variable to be imputed, seeking the donor within the same municipality. Thus, all variables used in this study have complete information. The details can be found elsewhere ^31^.

### Indicators of use of micronutrient supplements

For all constructed indicators, “micronutrient supplement use” was considered if the child was consuming it or had consumed it in the six months prior to the interview. The magnitude of micronutrient supplements use was described based on the variables “supplement use” (yes or no) and “number of supplements consumed” (1, 2, 3, or more).

The details of the composition of the supplements included iron, vitamin A, vitamin C, and vitamin D. These micronutrients were chosen for the following reasons: (a) they are the target of public policies in Brazil (iron and vitamin A) ^32^ ^,^ ^33^, (b) are present in technical recommendations (vitamin D) ^15^ ^,^ ^17^, (c) are often the target of advertising (vitamin C) ^34^, or (d) are recurrently consumed (multivitamin) ^35^. In addition to these nutrients, calcium, zinc, vitamins K, E, and B-complex vitamins were also included in the characterization of the multivitamins, as they were part of the composition of the most prevalent supplements in this category.

The products were divided into five groups: supplements containing a single micronutrient; PNSF supplements; multivitamin supplements with or without minerals; multivitamin supplements without minerals including a description of the composition of the supplement or group of supplements more frequently consumed; and multivitamin supplements with minerals also including a description of the composition of the supplement or group of supplements more frequently consumed. The variable “supplements containing only vitamin A” includes vitamin A megadose (PNSVA), and the variable “multivitamin supplements with minerals” includes NutriSUS. The variable “PNSF supplement” was constructed based on information about using a supplement with only iron acquired via SUS.

### Data analysis

Prevalence estimates and their respective 95% confidence intervals (95%CI) were calculated to characterize the micronutrient supplements use by Brazilian children 6-59 months of age and were stratified by age group (6-23 and 24-59 months of age). The estimates were calculated for Brazil and according to the macroregion (North, Northeast, Southeast, South, and Central-West), education level of the mother or caregiver (0-7, 8-10, 11, and ≥ 12 years of study), and type of health care service used (SUS user or SUS non-user). This last information was extracted from the question about the place where the child used to be taken for a medical appointment most frequently. The classification of the responses considered as “SUS users” were those who reported “basic health unit”, “specialty center, public polyclinic, or medical center”, or “public hospital/outpatient clinic”. The other options, which included “other”, “I do not usually take my child for medical appointments”, “private office or private clinic”, and “outpatient clinic or office of a company or union”, were considered in the category “SUS non-users”.

The coefficient of variation is a measure of dispersion that indicates the heterogeneity of the data, obtained by the ratio between the standard error and the estimated value of the indicator multiplied by 100 to estimate the percentage of variation. Estimates that have a coefficient of variation ≥ 30% may indicate that the sample is not large enough to perform the estimation at the population level with an acceptable degree of accuracy and should be interpreted with caution. A coefficient of variation of ≤ 30% was established as a good level of accuracy in this study. It was considered that the difference between proportions was statistically significant when there was no overlap of the 95%CI of the point estimates.

All analyses were performed via the R (http://www.r-project.org) programming language using the functions of the packages *srvyr* and *survey* to consider the structure of the sampling plan, the weights, and the calibration.

### Ethical considerations

The ENANI-2019 was approved by the Research Ethics Committee of the Clementino Fraga Filho University Hospital of the Federal University of Rio de Janeiro (UFRJ; CAAE n. 89798718.7.0000.5257). Data were collected after a parent or caregiver of the child authorized participation in the study through informed consent form.

## Results

Most children studied (54.2%, 95%CI: 50.5; 57.8) 6-59 months of age consumed (or had consumed in the 6 months preceding the study) micronutrient supplements, and 22% were consuming (or had consumed) 2 or more supplements. Among the group of supplements containing a single micronutrient, for children 6-59 months of age, the use of vitamin A had the highest prevalence (23.3%, 95%CI: 19.4; 27.1), followed by iron (14.6%, 95%CI: 13.1; 16.1), and vitamin C (13%, 95%CI: 10.4; 15.5) (Tables 1 and 2).

The use of multivitamin supplements with or without minerals was observed for 24.3% (95%CI: 21.4; 27.2) of the children, and 16.1% (95%CI: 13.9; 18.3) of them consumed multivitamins without minerals, 9.2% (95%CI: 7.1; 11.3) multivitamins with minerals, and 1% (95%CI: 0.7; 1.3) consumed more than one multivitamin. The most prevalent multivitamins without minerals were those containing vitamins A, C, D, E, and B complex (4.9%, 95%CI: 3.8; 6.0); with only vitamins A and D (4.6%, 95%CI: 3.4; 5.9); and with only vitamins C and B complex (4.3%, 95%CI: 3.3; 5.2). For multivitamins with minerals, the most prevalent supplement contained vitamins A, D, E, and B complex, iron, zinc, copper, selenium, and iodine (2.4%, 95%CI: 0.7; 4.2) with no significant difference (Table 1).

**Table 1:** Prevalence of micronutrient supplements use by age group among Brazilian children. *Brazilian National Survey on Child Nutrition* (ENANI-2019).

Supplements	Age group (months)					
	6-59		6-23		24-59	
	%	95%CI	%	95%CI	%	95%CI
Supplements use	54.2	50.5; 57.8	69.5	65.7; 73.3	46.5	42.4; 50.5
Number of supplements consumed						
1	32.2	29.9; 34.4	35.8	32.6; 39.0	30.3	27.6; 33.0
2	16.8	14.9; 18.7	25.0	22.9; 27.2	12.7	10.5; 14.9
≥ 3	5.2	3.9; 6.4	8.7	6.7; 10.6	3.4	2.3; 4.6
Supplements containing only iron *	14.6	13.1; 16.1	29.7	26.1; 33.2	7.1	5.9; 8.3
PNSF supplements	5.9	4.7; 7.2	11.5	8.8; 14.3	3.1	2.1; 4.0
Supplements containing only vitamin						
A **	23.3	19.4; 27.1	25.5	20.8; 30.1	22.2	18.4; 25.9
C	13.0	10.4; 15.5	15.9	12.4; 19.4	11.5	9.2; 13.8
D	3.2	2.2; 4.1	7.8	5.3; 10.3	0.8	0.4; 1.2
Multivitamin supplements with or without minerals ***	24.3	21.4; 27.2	30.1	26.0; 34.3	21.4	17.9; 24.9
Multivitamin supplements without minerals	16.1	13.9; 18.3	22.4	19.1; 25.6	13.0	10.7; 15.3
Vitamins A, C, D, E, and B complex	4.9	3.8; 6.0	7.1	5.3; 8.9	3.9	2.7; 5.0
Vitamins A and D	4.6	3.4; 5.9	11.7	8.4; 15.0	1.1	0.6; 1.5
Vitamins C and B complex	4.3	3.3; 5.2	1.3	0.8; 1.9	5.7	4.4; 7.1
Vitamins A, C, D, and B complex	1.4	1.0; 1.8	0.9	0.4; 1.4	1.7	1.1; 2.2
Vitamins A, D and E	0.5	0.3; 0.7	1.0	0.5; 1.5	0.2 ^#^	0.0; 0.4
B complex	0.5 ^#^	0.2; 0.9	0.3 ^#^	0.0; 0.5	0.7 ^#^	0.3; 1.1
Multivitamin supplements with minerals ***	9.2	7.1; 11.3	9.1	7.0; 11.2	9.2	6.5; 12.0
Vitamins A, D, E, and B complex, iron, zinc, copper, selenium, iodine ***	2.4	0.7; 4.2	0.5	0.2; 0.8	3.4	0.8; 6.1
Vitamins C e D, iron and calcium	1.8	1.0; 2.6	1.4 ^#^	0.3; 2.5	2.0	1.1; 2.9
Vitamins A, C, D and B complex, iron, zinc	1.0	0.7; 1.3	1.5	09; 2.1	0.7	0.3; 1.0
B complex and iron	0.7 ^#^	0.4; 1.0	1.2 ^#^	0.4; 2.0	0.4 ^#^	0.1; 0.7
B complex, Vitamin D, calcium and zinc	0.5	0.3; 0.7	1.1	0.5; 1.7	0.2 ^#^	0.1; 0.4

95%CI: 95% confidence interval.

* Includes information on supplements use from the Brazilian National Iron Supplementation Program (PNSF);

** Includes information on supplements use from the Brazilian National Vitamin A Supplementation Program (PNSVA);

*** Includes information on the use of the Brazilian National Strategy of Fortification of Infant Feeding with Micronutrients in Powder (NutriSUS);

^#^ Coefficients of variation ≥ 30%. The coefficients of variation is a measure of dispersion that indicates the heterogeneity of the data obtained by the ratio between the standard error and the estimated value of the indicator.

There was a higher prevalence of almost all supplements - or groups of supplements - among children 6-23 months of age compared to children 24-59 months of age (Table 1). Specifically, the prevalence of PNSF supplements use among children 6-23 months of age (target group of the program) was 11.5% (95%CI: 8.8; 14.3) (Table 1). In this age group, the highest prevalence of use of these supplements was observed in the Southeast Region (21.6%, 95%CI: 14.8; 28.3), in the group of mothers or caregivers with 0-7 years of study (14.5%, 95%CI: 11.3; 17.6), and among SUS users (14.1%, 95%CI: 10.6; 17.5) (significant differences to other category or categories of each of these three variables) (Table 3).

The highest prevalence of the use of supplements and the use of 2 or more supplements were observed in the North Region (80.2% and 37%, respectively). The lowest prevalence was in the South Region (27.5% and 8.4%, respectively) for children 6-59 months of age (Table 2) and according to the age group (Tables 3 and 4) (significant differences). No significant differences were found between the prevalence of these indicators regarding the level of education of the mother or caregiver or the type of health care service used (Tables 2, 3, and 4).

**Table 2:** Prevalence of micronutrient supplements use among children aged 6-59 months in Brazil and according to sociodemographic characteristics. *Brazilian National Survey on Child Nutrition* (ENANI-2019).

Variables	Supplement use	Number of supplements used			Supplements containing iron *	PNSF supplements
		1	2	≥ 3		
	% (95%CI)	% (95%CI)	% (95%CI)	% (95%CI)	% (95%CI)	% (95%CI)
Brazil	54.2 (50.5; 57.8)	32.2 (29.9; 34.4)	16.8 (14.9; 18.7)	5.2 (3.9; 6.4)	14;6 (13.1; 16.1)	5.9 (4.7; 7.2)
Macroregion						
North	80.2 (74.9; 85.6)	43.2 (39.8; 46.7)	27.6 (27.6; 31.6)	9.4 (6.1; 12.8)	10.2 (7.0; 13.4)	2.6 (1.5; 3.7)
Northeast	69.9 (60.9; 78.9)	35.4 (31.8; 38.9)	25.9 (20.1; 31.7)	8.6 (4.9; 12.4)	11.0 (8.8; 13.1)	1.9 (1.1; 2.6)
Southeast	44.7 (38.6; 50.9)	30.3 (25.5; 35.1)	11.3 (9.4; 13.2)	3.1 (1.8; 4.5)	19.1 (15.9; 22.3)	10.5 (7.5; 13.6)
South	27.5 (22.9; 32.0)	19.2 (15.5; 22.9)	7.4 (5.4; 9.3)	1.0 (0.4; 1.5)	12.5 (10.1; 15.0)	4.1 (2.8; 5.4)
Central-West	54.4 (47.7; 61.1)	36.7 (31.8; 41.5)	13.2 (10.3; 16.2)	4.5 (2.8; 6.3)	14.9 (12.0; 17.9)	5.1 (3.0; 7.2)
Mother or caregiver education level (years of study)						
0-7	49.3 (43.5; 55.2)	31.0 (26.5; 35.4)	14.3 (11.9; 16.8)	4.0 (2.6; 5.4)	12.4 (9.8; 14.9)	6.5 (4.4; 8.5)
8-10	53.2 (48.0; 58.5)	33.1 (29.1; 37.1)	15.7 (12.2; 19.1)	4.5 (2.8; 6.1)	11.5 (8.6; 14.4)	6.9 (4.1; 9.7)
11	57.2 (53.1; 61.4)	32.2 (29.5; 36.9)	18.8 (16.4; 21.3)	5.2 (3.9; 6.5)	17.3 (15.0; 19.5)	6.2 (4.3; 8.1)
≥ 12	54.7 (49.9; 59.4)	30.2 (27.2; 33.2)	16.8 (14.2; 19.4)	7.7 (4.3; 11.1)	15.4 (12.0; 18.9)	3.1 (1.4; 4.8)
Health care service used						
SUS user	53.7 (49.6; 57.9)	32.4 (29.7; 35.2)	16.2 (14.0; 18.4)	5.1 (3.8; 6.4)	14.3 (12.5; 16.0)	7.0 (5.4; 8.6)
SUS non-user	55.8 (51.3; 60.4)	31.1 (27.9; 34.4)	19.2 (16.0; 22.3)	5.5 (3.6; 7.5)	16.0 (13.0; 19.0)	1.5 (0.6; 2.5) **
Variables	Supplements containing only vitamin				Multivitamin supplements with or whithout minerals ***	
	A ^#^	PNSVA	C	D		
	% (95%CI)	% (95%CI)	% (95%CI)	% (95%CI)		
Brazil	23.3 (19.4; 27.1)	23.1 (19.2; 26.9)	13.0 (10.4; 15.5)	3.2 (2.2; 4.1)	24.3 (21.4; 27.2)	
Macroregion						
North	43.3 (32.0; 54.6)	42.6 (31.5; 53.7)	31.8 (23.3; 40.3)	3.9 ** (0.4; 7.5)	32.4 (25.5; 39.3)	
Northeast	45.8 (35.2; 56.3)	45.7 (35.1; 56.2)	21.1 (13.6; 28.7)	4.2 (2.7; 5.8)	27.5 (20.8; 34.1)	
Southeast	7.8 ** (2.7; 12.9)	7.7 ** (2.6; 12.7)	6.1 (3.7; 8.6)	1.9 ** (0.2; 3.6)	25.0 (19.8; 30.2)	
South	1.9 ** (0.6; 3.2)	1.5 ** (0.2; 2.9)	2.5 (1.2; 3.8)	4.2 (2.3; 6.2)	13.6 (10.5; 16.7)	
Central-West	28.6 (20.1; 37.2)	28.5 (20.0; 37.0)	9.8 (7.6; 11.9)	2.9 (1.8; 3.9)	17.4 (15.3; 19.6)	
Mother or caregiver education level (years of study)						
0-7	25.6 (20.6; 30.5)	25.2 (20.2; 30.2)	10.4 (7.4; 13.3)	1.1 (0.6; 1.6)	19.7 (15.6; 23.7)	
8-10	27.0 (21.1; 32.8)	26.9 (21.1; 32.7)	12.5 (9.3; 15.7)	1.9 (1.0; 2.7)	22.6 (18.9; 26.4)	
11	23.1 (18.9; 27.2)	22.8 (18.7; 27.0)	13.9 (10.9; 17.0)	3.8 (1.8; 5.7)	25.4 (22.1; 28.7)	
≥ 12	15.9 (10.5; 21.4)	15.9 (10.4; 21.3)	14.8 (10.7; 18.9)	6.2 (4.6; 7.7)	30.3 (25.6; 35.1)	
Health care service used						
SUS user	25.0 (20.7; 29.3)	24.8 (20.5; 29.0)	12.5 (10.0; 15.1)	2.4 (1.4; 3.3)	23.2 (20.0; 26.5)	
SUS non-user	16.3 (12.5; 20.1)	16.2 (12.4; 20.0)	14.7 (11.1; 18.3)	6.3 (4.5; 8.1)	28.8	(25.2; 32.4)

95%CI: 95% confidence interval; SUS: Brazilian Unified National Health System.

* Includes information on supplements use from the Brazilian National Iron Supplementation Program (PNSF);

** Coefficients of variation ≥ 30%. The coefficients of variation is a measure of dispersion that indicates the heterogeneity of the data obtained by the ratio between the standard error and the estimated value of the indicator;

*** Includes information on the use of the Brazilian National Strategy of Fortification of Infant Feeding with Micronutrients in Powder (NutriSUS);

^#^ Includes information on supplements use from the Brazilian National Vitamin A Supplementation Program (PNSVA).

**Table 3:** Prevalence of micronutrient supplements use among children aged 6-23 months in Brazil and according to sociodemographic characteristics. *Brazilian National Survey on Child Nutrition* (ENANI-2019).

Variables	Supplement use	Number of supplements used			Supplements containing iron *	PNSF supplements
		1	2	≥ 3		
	% (95%CI)	% (95%CI)	% (95%CI)	% (95%CI)	% (95%CI)	% (95%CI)
Brazil	69.5 (65.7; 73.3)	35.8 (32.6; 39.0)	25.0 (22.9; 27.2)	8.7 (6.7; 10.6)	29.7 (26.1; 33.2)	11.5 (8.8; 14.3)
Macroregion						
North	87.2 (79.4; 94.9)	42.9 (37.2; 48.6)	28.9 (24.6; 33.2)	15.4 (9.7; 21.0)	16.6 (9.5; 23.8)	3.5 ** (0.8; 6.2)
Northeast	78.9 (70.9; 86.9)	34.5 (29.3; 39.6)	30.2 (25.0; 35.4)	14.3 (9.7; 18.8)	21.5 (16.5; 26.5)	3.0 (1.7; 4.3)
Southeast	65.9 (59.1; 72.8)	36.0 (29.5; 42.5)	24.3 (21.0; 27.6)	5.7 (2.4; 9.0)	40.3 (32.7; 47.9)	21.6 (14.8; 28.3)
South	50.8 (42.2; 59.3)	31.3 (23.8; 38.8)	17.5 (12.3; 22.6)	2.0 (0.6; 3.4)	29.4 (22.9; 35.9)	10.4 (6.9; 14.0)
Central-West	61.2 (53.3; 69.2)	37.6 (3.3; 43.9)	17.8 (13.7; 21.9)	5.8 (2.5; 9.1)	24.8 (20.8; 28.7)	5.9 (3.5; 8.3)
Mother or caregiver education level (years of study)						
0-7	63.8 (57.6; 70.1)	35.4 (30.0; 40.9)	22.3 (17.3; 27.3)	6.1 (3.2; 9.0)	25.9 (20.4; 31.5)	14.5 (11.3; 17.6)
8-10	62.5 (56.0; 69.0)	34.6 (28.4; 40.8)	21.4 (16.2; 26.6)	6.5 (3.5; 9.6)	21.4 (16.0; 26.8)	12.0 (6.9; 17.1)
11	74.4 (70.4; 78.9)	37.0 (31.3; 42.6)	28.7 (24.2; 33.1)	9.1 (6.4; 11.7)	34.9 (28.3; 41.6)	12.2 (6.5; 17.8)
≥ 12	74.1 (67.8; 80.5)	35.3 (29.1; 41.6)	25.1 (19.9; 30.4)	13.7 (8.0; 19.3)	33.4 (26.6; 40.2)	6.3 (3.0; 9.6)
Health care service used						
SUS user	67.9 (63.8; 72.0)	35.4 (31.9; 39.0)	23.9 (21.2; 26.7)	8.5 (6.3; 10.7)	28.3 (24.9; 31.8)	14.1 (10.6; 17.5)
SUS non-user	75.7 (69.6; 81.9)	37.2 (31.6; 42.7)	29.2 (23.6; 34.7)	9.4 (5.8; 13.0)	34.8 (27.7; 41.8)	1.7 ** (0.0; 3.6)
Variables	Supplements containing only vitamin				Multivitamin supplements with or whithout minerals ***	
	A ^#^	PNSVA	C	D		
	% (95%CI)	% (95%CI)	% (95%CI)	% (95%CI)		
Brazil	25.5 (20.8; 30.1)	25.2 (20.5; 29.8)	15.9 (12.4; 19.4)	7.8 (5.3; 10.3)	30.1 (26.0; 34.3)	
Macroregion						
North	43.2 (28.5; 57.9)	43.2 (28.5; 57.9)	38.6 (28.6; 48.6)	8.2 (0.7; 15.7)	36.7 ** (29.7; 43.7)	
Northeast	50.0 (38.0; 62.0)	49.9 (37.8; 61.9)	27.2 (16.7; 37.7)	10.4 (6.9; 13.8)	27.0 (19.5; 34.4)	
Southeast	9.7 ** (3.0; 16.4)	9.3 ** (2.6; 16.1)	7.9 (4.2; 11.6)	5.2 (0.1; 10.3)	35.2 ** (26.7; 43.8)	
South	3.1 ** (0.9; 5.2)	2.5 ** (0.4; 4.6)	1.5 ** (0.2; 2.9)	11.0 (5.3; 16.6)	24.2 (16.3; 32.1)	
Central-West	29.2 (20.1; 38.3)	28.9 (19,9; 37.9)	8.6 (5.8; 11.3)	6.2 (3.7; 8.7)	17.8 (14.9; 20.8)	
Mother or caregiver education level (years of study)						
0-7	28.6 (22.1; 35.0)	27.7 (21.0; 34.4)	15.5 (9.7; 21.3)	2.7 (1.4; 4.1)	22.1 (17.4; 26.9)	
8-10	29.1 (22.7; 35.5)	29.0 (22.6; 35.4)	15.7 (10.1; 21.3)	3.6 (1.8; 5.5)	24.7 (19.0; 30.5)	
11	25.3 (19.3; 31.3)	25.2 (19.2; 31.2)	16.1 (11.5; 20.7)	9.8 (4.2; 15.3)	33.4 (27.5; 39.3)	
≥ 12	17.6 (10.0; 25.1)	17.3 (9.7; 24.9)	16.1 (9.5; 22.7)	15.2 (11.0; 19.4)	39.7 (32.7; 46.7)	
Health care service used						
SUS user	27.5 (22.4; 32.6)	27.2 (22.1; 32.3)	16.2 (12.1; 20.3)	6.3 (6.5; 9.0)	28.0 (23.6; 32.4)	
SUS non-user	17.6 (11.5; 23.8)	17.5 (11.3; 23.7)	14.8 (10.1; 19.4)	14.0 (9.8; 18.1)	38.4	(32.8; 44.0)

95%CI: 95% confidence interval; SUS: Brazilian Unified National Health System.

* Includes information on supplements use from the Brazilian National Iron Supplementation Program (PNSF);

** Coefficients of variation ≥ 30%. The coefficients of variation is a measure of dispersion that indicates the heterogeneity of the data obtained by the ratio between the standard error and the estimated value of the indicator;

*** Includes information on the use of the Brazilian National Strategy of Fortification of Infant Feeding with Micronutrients in Powder (NutriSUS);

^#^ Includes information on supplements use from the Brazilian National Vitamin A Supplementation Program (PNSVA).

**Table 4:** Prevalence of micronutrient supplements use among children aged 24-59 months in Brazil and according to sociodemographic characteristics. *Brazilian National Survey on Child Nutrition* (ENANI-2019).

Variables	Supplement use	Number of supplements used			Supplements containing iron *	PNSF supplements
		1	2	≥ 3		
	% (95%CI)	% (95%CI)	% (95%CI)	% (95%CI)	% (95%CI)	% (95%CI)
Brazil	46.5 (42.4; 50.5)	30.3 (27.6; 33.0)	12.7 (10.5; 14.9)	3.4 (2.3; 4.6)	7.1 (5.9; 8.3)	3.1 (2.1; 4.0)
Macroregion						
North	76.7 (71.2; 82.3)	43.4 (39.5; 47.3)	26.9 (21.7; 32.1)	6.4 (4.1; 8.8)	7.0 (4.3; 9.7)	2.1 (1.3; 3.0)
Northeast	65.4 (55.1; 75.7)	35.8 (30.9; 40.8)	23.8 (16.8; 30.7)	5.8 (2.1; 9.5)	5.6 (4.0; 7.3)	1.3 ** (0.5; 2.1)
Southeast	34.1 (27.2; 41.0)	27.5 (22.0; 32.9)	4.8 (2.7; 6.8)	1.9 (0.9; 2.8)	8.5 (6.0; 11.1)	5.0 (2.8; 7.3)
South	15.8 (11.5; 20.2)	13.1 (9.0; 17.2)	2.3 (1.1; 3.6)	0.4 (0.0; 0.9)	4.1 (2.6; 5.6)	0.9 (0.4; 1.5)
Central-West	51.0 (44.2; 57.8)	36.2 (31.2; 41.2)	10.9 (8.1; 13.7)	3.9 (2.1; 5.7)	10.0 (9.8; 13.1)	4.7 ** (1.9; 7.4)
Mother or caregiver education level (years of study)						
0-7	42.7 (35.7; 49.7)	28.9 (23.4; 34.5)	10.7 (8.1; 13.3)	3.1 (1.4; 4.7)	6.2 (3.7; 8.7)	2.9 ** (0.9; 4.9)
8-10	48.0 (42.1; 53.9)	32.3 (28.0; 36.5)	12.5 (8.9; 16.0)	3.3 (1.5; 5.1)	5.9 (4.1; 7.7)	4.1 (2.3; 5.8)
11	48.9 (44.2; 53.7)	31.4 (27.7; 35.1)	14.2 (11.6; 16.7)	3.4 (2.3; 4.5)	8.9 (6.7; 11.0)	3.4 (1.9; 4.9)
≥ 12	43.8 (38.3; 49.2)	27.2 (23.0; 31.5)	12.2 (8.7; 15.6)	4.3 (0.9; 7.8)	5.3 (2.9; 7.7)	1.3 ** (0.1; 2.6)
Health care service used						
SUS user	46.7 (42.0; 51.4)	30.9 (27.6; 34.3)	12.4 (10.0; 14.8)	3.4 (2.3; 4.6)	7.3 (5.7; 8.9)	3.5 (2.3; 4.6)
SUS non-user	45.4 (39.7; 51.2)	28.0 (24.1; 31.8)	14.0 (10.4; 17.5)	3.5 (1.5; 5.5)	6.2 (4.2; 8.2)	1.4 ** (0.2; 2.6)
Variables	Supplements containing only vitamin				Multivitamin supplements with or whithout minerals ***	
	A ^#^	PNSVA	C	D		
	% (95%CI)	% (95%CI)	% (95%CI)	% (95%CI)		
Brazil	22.2 (18.4; 25.9)	22.0 (18.3; 25.7)	11.5 (9.2; 13.8)	0.8 (0.4; 1.2)	21.4 (17.9; 24.9)	
Macroregion						
North	43.3 (32.7; 54.0)	42.3 (32.2; 52.5)	28.4 (20.1; 36.6)	1.8 ** (0.1; 3.5)	3.3 (22.8; 37.7)	
Northeast	43.6 (32.9; 54.3)	43.6 (32.9; 54.2)	18.1 (11.3; 24.8)	1.1 ** (0.0; 2.2)	27.7 (20.2; 35.2)	
Southeast	6.9 ** (2.2; 11.5)	6.8 ** (2.2; 11.5)	5.3 (2.8; 7.7)	0.2 ** (0.0; 0.5)	19.9 (13.2; 26.6)	
South	1.3 ** (0.2; 2.4)	1.1 ** (0.0; 2.1)	3.0 (1.4; 4.6)	0.8 ** (0.2; 1.5)	8.2 (5.3; 11.2)	
Central-West	28.3 (19.5; 37.2)	28.2 (19.4; 37.1)	10.4 (7.7; 13.1)	1.2 (0.6; 1.7)	17.2 (14.7; 19.8)	
Mother or caregiver education level (years of study)						
0-7	24.2 (18.8; 29.6)	24.1 (18.6; 29.5)	8.0 (5.5; 10.6)	0.3 ** (0.0; 0.7)	18.6 (13.3; 23.8)	
8-10	25.7 (19.3; 32.2)	25.7 (19.2; 32.2)	10.7 (7.8; 13.5)	0.9 ** (0.1; 1.7)	21.5 (17.0; 25.9)	
11	22.0 (18.2; 25.9)	21.7 (17.9; 25.5)	12.9 (10.0; 15.9)	0.9 ** (0.3; 1.5)	21.6 (17.4; 25.8)	
≥ 12	15.1 (10.0; 20.1)	15.0 (9.9; 20.1)	14.1 (9.9; 18.2)	1.2 (0.5; 1.8)	25.1 (19.6; 30.5)	
Health care service used						
SUS user	23.8 (19.5; 28.1)	23.6 (19.3; 27.8)	10.7 (8.5; 12.9)	0.4 (0.2; 0.7)	20.8 (16.8; 24.9)	
SUS non-user	15.6 (11.9; 19.2)	15.6 (11.9; 19.2)	14.7 (10.4; 19.0)	2.3 ** (0.7; 3.9)	23.8	(19.7; 27.8)

95%CI: 95% confidence interval; SUS: Brazilian Unified National Health System.

* Includes information on supplements use from the Brazilian National Iron Supplementation Program (PNSF);

** Coefficients of variation ≥ 30%. The coefficients of variation is a measure of dispersion that indicates the heterogeneity of the data obtained by the ratio between the standard error and the estimated value of the indicator;

*** Includes information on the use of the Brazilian National Strategy of Fortification of Infant Feeding with Micronutrients in Powder (NutriSUS);

^#^ Includes information on supplements use from the Brazilian National Vitamin A Supplementation Program (PNSVA).

For children 6-59 months of age, supplements containing only iron showed a higher prevalence of use in the Southeast Region (19.1%, 95%CI: 15.9; 22.3) and among children of mother or caregiver with 11 years of education (17.3%) (significant differences to other categories of each of these variables) (Table 2). A similar scenario was observed with a higher prevalence for children 6-23 months of age (Table 3). Among children ≥ 24 months of age, the highest prevalence of use was observed in the Central-West (10%, 95%CI: 6.8; 13.1) (significantly different to the South Region). For this group, no significant difference was found between the prevalence observed according to the education level of the mother or caregiver and the type of health care service used (Table 4).

For the total sample and the two age groups analyzed, when examining the use of supplements containing only vitamin A, higher prevalence rates were observed in the North, Northeast, and Central-West regions (significant differences to the other areas) and among SUS users (with a significant difference in the sample of 6-59 months of age) (Tables 2, 3, and 4). Regarding supplements containing only vitamin C, for the total sample and both age groups, a significantly higher prevalence was observed in the North and Northeast regions (ranging from 18.1% to 38.6%) than in the other regions (ranging from 1.5% to 10.4%). No differences were observed according to the education level of the mother or caregiver and the type of health care service used. A significantly higher prevalence of the use of supplements containing only vitamin D was observed among children of mothers or caregivers with 11 and ≥ 12 years of education compared to 0-7, and SUS non-users in the total sample and among children < 2 years of age (Tables 2 and 3).

The use of multivitamins with or without minerals was more frequent in the North, Northeast, and Southeast regions compared to the South Region in the total sample and children ≥ 24 months of age, among children of mothers or caregivers with higher education levels in the total sample (≥ 12 vs. 0-7 years of education) and in children < 24 months of age (11 and ≥ 12 vs. 0-7 years of education) and among non-users of the SUS in children < 24 months of age (Tables 2, 3 and 4). The prevalence of NutriSUS vitamins use was 2.4% (95%CI: 0.7; 4.2), 0.5% (95%CI: 0.2; 0.8), and 3.4% (95%CI: 0.8; 6.1) for children 6-59, < 24 and those aged ≥ 24 months of age, respectively (data not shown in tables).

## Discussion

These results show a high prevalence of supplements use in the study group, which was higher among children 6-23 months of age. Heterogeneity was observed between the prevalence according to macroregion, education level of the mother or caregiver, or type of health care service used depending on the supplement considered. In addition to recording the use of products recommended and distributed by the Brazilian Ministry of Health (such as supplements containing exclusively iron or exclusively vitamin A and NutriSUS), the use of multivitamin supplements with or without minerals, supplements containing only vitamin C, and supplements containing only vitamin D were also documented.

In Brazil, the *National Survey on Access, Use and Promotion of Rational Use of Medicines* (PNAUM), conducted from 2013 to 2014, observed that 6.1% of participants > 12 years old had consumed some of these products in the 15 days before the study. The PNAUM also showed that ferrous sulfate and multivitamins were among the 10 most commonly consumed products by children 24-59 months of age (4.9% and 5%, respectively) ^20^.

In ENANI-2019, a group of children consumed more than one supplement during the study period: 1.1% (95%CI: 0.7; 1.6) consumed vitamin A megadose and PNSF supplements, and 14.5% (95%CI: 11.8; 17.3) consumed one of these supplements and another product that is not part of public policies. Healthcare professionals should carefully prescribe micronutrient supplements for children. Knowing the supplements being used by the chid and adapting the prescription to the child’s need is necessary to avoid overlapping supplements, overdoses, and adverse reactions.

Among the supplements containing a single micronutrient, the highest prevalence of vitamin A use was observed among children 6-59 months of age. The use of PNSVA supplements corresponds to approximately 99% of the reported use of vitamin A-only supplements. Initially directed to children 6-59 months of age in the Northeast Region, in the Vale do Jequitinhonha (Minas Gerais State) and in the Vale do Ribeira (São Paulo State), PNSVA scope was expanded over the years to cover other territories potentially vulnerable to vitamin A deficiency ^22^ ^,^ ^36^ ^,^ ^37^. The PNDS 2006 showed that 30.7% of Brazilian children should receive vitamin A supplementation and that 94% of them had received it ^22^. However, according to the Brazilian Ministry of Health, approximately 51% of the PNSVA coverage target was reached in 2017 ^38^. Although the results of ENANI-2019 do not allow for a refined analysis of this coverage, they indicate a higher prevalence of the use of supplements containing only vitamin A in the North and Northeast regions among children with a mother or caregiver with a lower level of education and among SUS users, which converges with the design of the program.

Prophylactic iron supplementation, one of the strategies of the Brazilian Ministry of Health to control anemia, should be universal for children 6-23 months of age ^33^. The results of ENANI-2019 show that PNSF coverage is still low. Even considering the use of multivitamins with iron, which was 7.9% (95%CI: 5.9; 9.9) for those 6-23 months of age (data not shown in the *Results*), the prevalence of iron supplementation is much lower than the desired universal coverage. This scenario is even more problematic in the North Region, where the use of supplements containing only iron, iron supplements through the PNSF, and multivitamins with iron (15.8%, 95%CI: 10.3; 21.2) are low, and where in 2019, the highest prevalence of anemia (30.3%) and iron-deficiency anemia (13.9%) was recorded within this age group (data not shown in the *Results*) ^39^. The low prevalence of PNSF supplements use can be explained, at least partly, by a low adherence of users, conduct of insufficient encouragement and/or inadequate guidance by professionals with families, and the decentralization of the program in 2013, which may have resulted in a less-efficient provision of the supplements ^40^ ^,^ ^41^ ^,^ ^42^.

In the PNAUM, the prevalence of iron-fortified salt was 8.5% among children < 12 months of age and 5.6% among children 12-23 months of age, with the lowest prevalence recorded in the Central-West and South regions ^20^. The results of the *Brazilian National Health Survey* 2013 (PNS 2013) showed that 57.9% of children 6-23 months of age received ferrous sulfate at some point in life, with the Southeast Region having the highest (69.9%) and the North Region the lowest prevalence (40.3%) ^43^. These results should be cautiously compared, given the methodological differences regarding the age range of the children studied and the period of supplements use considered at the interview.

Supplements containing vitamins C and D, which are not the target of specific programs, were frequently consumed by the children evaluated in ENANI-2019, either alone or in multivitamins with or without minerals (18.1%, 95%CI: 15.6; 20.6 and 18.7%, 95%CI: 16.2; 21.3, respectively), respectively. In childhood, vitamin C supplement use is recommended by the Brazilian Ministry of Health at a 30mg/day dose only for children < 4 months of age who are not breastfed and receive cow’s milk. Notably, supplementation is not necessary for those receiving infant formula ^44^ ^,^ ^45^. Vitamin C can improve iron absorption for children with low iron use. Families often use vitamin C without a prescription for preventing and treating colds, an effect whose efficacy has not been proven ^20^ ^,^ ^46^. The daily requirements of vitamin C can be easily met with food, i.e., supplementation is only justified in specific cases, as the Brazilian Ministry of Health recommends. According to the PNAUM results, vitamin C was the fifth most consumed product among individuals < 12 years old ^20^. It is noteworthy that consuming vitamin C above the recommended daily limit may cause adverse effects, such as gastrointestinal symptoms and the formation of kidney stones ^47^.

In Brazil, the prevalence of vitamin D insufficiency is very low among children 6-59 months of age (4.3%) ^39^, and there is no public policy aimed at supplementing this micronutrient. Nevertheless, the Brazilian Society of Pediatrics recommends vitamin D supplementation for newborn infants exclusively breastfed or who are fed enriched-infant formula in amounts < 1,000mL/day to prevent vitamin D deficiency ^15^ ^,^ ^17^. However, approximately 80% to 90% of vitamin D obtained by the body comes from skin synthesis through exposure to UVB radiation ^48^, which may contribute to an adequate vitamin D status, especially in a tropical country such as Brazil. Thus, the *Dietary Guidelines for Children Under Two Years of Age* ^49^ does not guide vitamin D supplementation and emphasize the importance of exposing the child to the sun as it is the main form of vitamin D synthesis.

The number of vitamin D-based supplements available on the market has increased considerably, with several targeting children ^50^. The adverse effects of unnecessary vitamin D supplementation have already been recorded, with many reports of cases of intoxication among children. Errors in the formulation of the supplement have aggravated cases of intoxication, with some children ingesting 1,000-4,000 times the indicated dose and presenting side effects such as hypercalcemia, vomiting, weight loss, and constipation ^51^ ^,^ ^52^.

An important finding of ENANI-2019 is that 25% of Brazilian children 6-59 months of age consumed multivitamin supplements with or without minerals. The results from the PNAUM (2013-2014) showed that 45.6% of individuals > 12 years old consumed multivitamins with minerals ^53^. In ENANI-2019, the prevalence of use was higher among younger children (6-23 months of age), non-users of the SUS, and those with a mother or caregiver with a higher level of education. Knowing that education can be considered a proxy for income and, consequently, access to a healthy and varied diet, the real need for children to use these products should be questioned. There was heterogeneity in the prevalence of use of these products among the macroregions of Brazil; the supplements were more frequently consumed by children in the North Region and less frequently by children in the Central-West. These consumption dynamics may reflect different distribution strategies for these products and encouragement of their use by private or public healthcare services.

Among multivitamins without minerals, a considerable number contain B-complex vitamins and vitamins A, C, D, and E in their compositions, while among multivitamins with minerals, the most commonly used multivitamins contain vitamins A, D, E, and the B-complex vitamins, iron, zinc, copper, selenium, and iodine. The composition of multivitamins with or without minerals varies greatly, and micronutrient combinations are not necessarily based on scientific evidence. Regarding NutriSUS, the low prevalence of use of this supplements may be due to the low coverage of daycare centers (where the product was distributed at the time of the study) and also to the temporary discontinuation of supply of the product by the Brazilian Ministry of Health during the period of data collection of the study ^54^. 

Micronutrient supplements use aims to prevent or treat nutritional problems. However, use above safe levels can cause harm to health ^54^. A study conducted in the United States (2003-2006) observed that children 2-8 years old who consumed a micronutrient supplement more easily exceeded their maximum tolerable use limit ^55^. Therefore, given the scenario of supplements use observed in ENANI-2019, complementary analyses of the contribution of micronutrients from supplements and food are necessary to ascertain their adequacy.

The pioneering spirit of ENANI-2019 brought challenges to its concretion. From collection to analysis of the results, the insufficiency of information on data processing models and the absence of clear information on some products hindered the process, especially concerning obtaining accurate information on the composition and concentration of nutrients in the supplements, since there is no requirement for a package insert for these products ^29^ ^,^ ^56^.

The ENANI-2019 showed a high prevalence of micronutrient supplements use among children 6-59 months of age, with regional differences, educational level of the mother or caregiver, and type of health care service used depending on the supplement considered. It also showed, on the one hand, low coverage of the iron supplementation program and, on the other hand, a significant use of supplements that are not part of the Brazilian Ministry of Health programs.

This study is pioneer in describing the detailed record of supplements use among Brazilian children 6-59 months of age, providing novel and relevant information not only for the analysis of public policies of micronutrient supplementation implemented in Brazil but also for the problems resulting from unnecessary and indiscriminate use of these products.
